# Assessing Knowledge Gaps of Women and Healthcare Providers Concerning Cardiovascular Risk After Hypertensive Disorders of Pregnancy—A Scoping Review

**DOI:** 10.3389/fcvm.2019.00178

**Published:** 2019-11-29

**Authors:** Heike Roth, Grace LeMarquand, Amanda Henry, Caroline Homer

**Affiliations:** ^1^Faculty of Health, University of Technology Sydney, Sydney, NSW, Australia; ^2^School of Women's and Children's Health, UNSW Medicine, University of NSW, Sydney, NSW, Australia; ^3^Global Women's Health Program, The George Institute for Global Health, Sydney, NSW, Australia; ^4^Department of Women's and Children's Health, St George Hospital, Sydney, NSW, Australia; ^5^Burnet Institute, Maternal and Child Health, Melbourne, VIC, Australia

**Keywords:** knowledge, women, healthcare providers, preeclampsia, hypertension, cardiovascular risk

## Abstract

**Background:** A history of a Hypertensive Disorder of Pregnancy (HDP) at least doubles a woman's risk of cardiovascular disease (CVD). The risk increases within 10 years after HDP and continues for life, making long-term health after HDP of major public health importance. Understanding knowledge gaps in health care professionals and women regarding cardiovascular health after HDP is an important component in addressing these risks.

**Objectives:** The primary aim was to examine what women and healthcare providers (HCP) know about cardiovascular risks after HDP. The secondary aims were to identify enablers and barriers to knowledge and action on knowledge.

**Methods:** A scoping review was conducted. This was a narrative synthesis, using PRISMA-ScR guidelines, of English-language full text articles that included assessment of knowledge of women, and/or HCP, on long term cardiovascular risk after HDP. The databases Embase, Medline, Scopus, ProQuest, Cochrane, and PsycInfo were searched from 01 January 2005 to 31 May 2019.

**Results:** Twelve studies were included, six addressing women's knowledge, five addressing HCP knowledge, and one addressing both. The studies included 402 women and 1,215 HCP from seven countries. Regarding women's knowledge, six of seven studies found women had limited or no knowledge about the link between HDP and CVD. Where women were aware of the link, the majority had sourced their own information, rather than obtaining it through their HCP. In five of six studies, HCP also mostly had limited knowledge about HDP-CVD links. Primary enablers for HCP acquisition of knowledge and counseling were the availability and knowledge of guidelines. Where comparisons between HCP groups were made, obstetricians had greater knowledge than family physicians, internal medical specialists, or midwives.

**Conclusion:** There was a low level of knowledge amongst HCP and women about increased CVD risk after HDP. Where women had higher levels of knowledge, the information was often obtained informally rather than from HCP. There were variations in knowledge of HCP, with obstetricians generally more aware than other professions. Further country and context-specific research on current status of women's and HCP's knowledge is therefore necessary when creating educational strategies to address knowledge gaps after HDP.

## Introduction

Preeclampsia (PE) is a multi-system disorder unique to human pregnancy characterized by hypertension and involvement of one or more other organ systems and/or the fetus (1). Preeclampsia is well-recognized as a major cause of poor pregnancy outcome. It is one of the top three causes of maternal mortality and severe morbidity in both high and low resource countries, leading directly to over 50,000 maternal deaths globally per year (2). For babies, up to one in five premature births occur following their mothers having preeclamptic pregnancies (3).

In addition to the short-term impacts, long-term adverse maternal health outcomes after preeclampsia and other Hypertensive Disorders of Pregnancy (HDP) may be an even greater burden of disease. Cardiovascular disease (CVD), the leading cause of death in women globally (4), is 2–2.5 times higher for women who have experienced preeclampsia at some stage in their life compared with those who had normotensive (normal blood pressure) pregnancies (5–7). This risk of premature death is present 8–20 years after the affected pregnancy (6, 8, 9). Gestational Hypertension (GH), new-onset hypertension without any other complications during pregnancy, has little association with adverse pregnancy outcomes (1), however is associated with long-term cardiovascular sequela (7, 10). Together with essential hypertension (EH), which indicates pre-existing increased cardiovascular risk, these HDP conditions complicate ~10% of pregnancies (3).

Peak cardiovascular health organizations, such as the American Heart Foundation, now recommend health care providers ask women about their HDP history when assessing their cardiovascular health and risk factors. The International Society for the Study of Hypertension in Pregnancy (ISSHP) recommendations (5) address the postpartum management of HDP and recommend a review at 3 months to ensure screening tests are within normal range or alternatively ensure appropriate referral occurs. ISSHP also recommends informing women of their long-term CVD risk, adoption of a healthy lifestyle with maintenance of an ideal weight and regular aerobic exercise, and regular follow-up with a general practitioner to monitor blood pressure and periodic measurement of fasting lipids and blood sugar.

Despite these recommendations, clinicians may not be aware of the association between HDP and CVD, suggesting that women are not given appropriate information about health after HDP (11–16). Several studies conclude that both health care providers and women should be provided with information regarding the link between HDP and later CVD (17–19). Therefore, the primary aim of this paper was to undertake a scoping review to examine what women and healthcare providers (HCP) know about cardiovascular risks after HDP. Secondary aims were to identify the aspects of care that can be seen as enablers and barriers to knowledge and action on knowledge.

## Methods

A scoping review of published literature on knowledge of women and/or health care providers about CVD risk after preeclampsia or gestational hypertension was undertaken. Scoping reviews follow a systematic approach to identify main concepts, evidence, and knowledge gaps on a specific topic (20). This methodology was appropriate given our interest in the broad topic of knowledge on health after HDP and the likely heterogeneous nature of the body of literature. This scoping review adheres to PRISMA-ScR guidelines (20) (Appendix 1) and the CASP (21) qualitative checklist was also used to assess quality in the of included qualitative literature (Appendix 2). Narrative synthesis was applied to analyze the included literature.

We searched multiple Databases including Embase, Medline, Scopus, ProQuest, Cochrane, and PsycInfo. The year of publication was limited to 1st January 2005 to 31st May 2019. Key words descriptors, Medical Subject Headings as well as MeSH terms were (but not limited to): “Health Knowledge, Attitudes, Practice,” “Education^*^,” “Communication Barriers,” “Risk perception,” “Enablers,” “Knowledge,” “Knowledge gap,” “Knowledge sharing,” “Pre-eclampsia,” “gestational hypertension,” “Hypertension, Pregnancy-Induced,” “future cardiovascular disease,” “long-term cardiovascular risk” (Appendix 3).

Key words were developed and separated into search categories. One was knowledge/education/risk perception, another was PE/GH/HDP and a third was long-term CVD risk. Database searches were accompanied by hand searching reference lists and citations of all included studies to identify any additional, relevant studies.

Papers were included (Supplementary Table 1) if they were original research addressing knowledge assessments, communication and awareness of long-term increased CVD risk after PE (including HELLP and eclampsia) or GH, full-text, available in English and if published during the selected timeframe. The date limit was applied as most initial cohort studies showing increased risk of CVD after preeclampsia were not published until the early 2000s, and the first risk meta-analysis was not published until 2007 (22). Studies of any methodology (qualitative/quantitative/mixed methods), sample size and type were eligible for inclusion.

Papers were excluded (Supplementary Table 1) if they were a conference or research abstract only, a review article without novel research, or items (e.g., study protocol or trial registration) that pertained to planned or in-progress research without published results. Where abstracts matched our search criteria and topic but with no details on study method and results and no full-text associated publications were found, authors were contacted for further details. However, this did not yield further inclusions by the cut-off of 31st May 2019.

Research that concerned chronic hypertension only (without superimposed PE) was excluded, as chronic hypertension is recognized by healthcare professionals as conveying increased CVD risk. Additionally, papers with focus only on the application of lifestyle recommendations to reduce the CVD risk after experiencing a pregnancy with hypertensive disease were excluded.

All articles were independently reviewed for inclusion by two reviewers who read the title, abstract, and full text. Discussion between the two reviewers resolved discrepancies. After summarizing the included papers, the papers were split into two categories: women's knowledge, and HCP's knowledge.

The Critical Appraisal Skills Programme (CASP) (21) tool was used to assess quality of the included literature. Although this is a tool designed to assist in systematic review inclusions, this quality appraisal tool was helpful in the systematic approach to enquire and reason about the studies eligible within the boundaries of the inclusion/exclusion criteria. The CASP checklist addresses three areas when appraising literature to be included in a review, these are validity, results and clinical relevance.

## Results

Of 1,467 identified articles, 12 studies met inclusion criteria (Supplementary Figure 1). Supplementary Table 2 summarizes the characteristics of the studies included.

Out of the 12 studies, six addressed women's knowledge (11, 13–16, 23), five addressed HCP's knowledge (24–28) and one addressed both women and HCP (12). The studies include eight quantitative (surveys, chart reviews) (15, 16, 23–28) and four qualitative studies (11–14) (focus group, group interview, semi-structured interviews). Of the qualitative studies, most were assessed as moderate to high quality on CASP criteria and details are itemized in Appendix 2. In total, the studies collected information from 402 women to 1,215 HCP.

Five studies were conducted in the United States of America (USA) (13–15, 27, 28) two from Canada (12, 26) one each from Australia (23), Germany (25), Nigeria (24), Portugal (16), and the United Kingdom (UK) (29).

The included studies displayed various levels of focus on knowledge assessment of CVD after HDP. Eleven conducted surveys or interviews exploring the women's or HCP's knowledge specifically and more extensively. One study's focus was on exploring general follow up of women with a history of PE and women's knowledge of CVD risk factors in general but did include a single question regarding women's knowledge on the link between PE and future CVD (16).

### Women's Knowledge

Seven (including the study addressing both women and HCP) studies addressed women's knowledge (11–16, 23). Three were quantitative (15, 16, 23) and four were qualitative studies (11–14). Six studies focused on the exploration of women's knowledge (11–15, 26) while one (within a study with a different main focus) included a question about whether counseling of HDP link to CVD had occurred (16).

Six out of the seven studies found that women had limited or no knowledge about the link between HDP and CVD. In the USA, Seely et al. (13) found that in 20 women after PE, the “majority” were not aware of their HDP CVD risks. In another study in the USA, 10 of the 14 participating women (71%) were unaware of the link between PE and CVD (14). A survey of 78 women in Portugal found that nearly 70% were not counseled on the link between PE and CVD (16). There was some evidence that women's knowledge of future CVD risk perception differed according to the type of severity of their HDP. A study of 146 women in the USA (of which 52% were without severe features, 28% PE had severe features such as HELLP and Eclampsia and 20% had Chronic Hypertension alone) found that CVD risk awareness was higher in those with severe PE (65%) and chronic hypertension (75%) than those with PE without severe features (43%) (13, 15). In Canada, Hird et al. (12) reported that the five women in their study were either not at all informed or partially informed. When informed this was limited to two out of five women finding out about potential recurrence of PE in a subsequent pregnancy. Four of the five women were not advised to have any follow-up blood tests. Only one out of the five women was advised about her CVD risk. In the UK, Brown et al (11) found that five of their 12 participants could not recall the HDP-CVD risk being raised with their HCP. Of those who were counseled of their risk, it was found that especially those women without a family history of CVD did not perceive the risk to apply to them.

The one exception was the Australian study by Hutchesson et al. (23), where close to two thirds of 127 women with a recent history of PE (≤ 2 years post PE) had higher knowledge about certain aspects of future CVD risk (96% answered “true” for future risk of hypertension and 66% answered “true” for future risk of stroke).

#### Women's Knowledge in the Early Postpartum Period vs. Later

There were conflicting findings regarding whether women in the first few years after HDP had higher knowledge about future CVD risk than those 5 years or more post-pregnancy. In Australia, Hutchesson et al. found 67% of women with recent PE (≤ 2 years) were aware of future CVD risk. However, in the USA a focus group study of women who had preeclampsia <5 years ago, the majority of the 20 women did not know of their future CVD risk until they attended the focus groups (13). There were similar findings in another focus group study of 14 women (14) where most (10/14) of the women were unaware of the link between PE and future CVD.

#### Sources of Knowledge, Enablers, and Barriers to Knowledge Acquisition in Women

Three of the seven studies explored sources of knowledge acquisition by the women (12, 14, 23). These showed that women in general wanted information on their HDP and to understand more about its link to future CVD. For example, in Brown et al. (29), all 12 women interviewed with a history of PE wanted to receive more information on PE and future implications on health. Enablers and barriers to women's knowledge acquisition were also addressed by most of the studies.

Of the seven studies that enquired about women's perceptions of being given information, major themes were that women did not receive information, or felt they received insufficient information, from their healthcare practitioners about risks after HDP. For those who were aware of long-term risks prior to being surveyed or interviewed, this knowledge had often been self-acquired. Hutchesson et al. (23) undertook a cross-sectional survey with women who had a recent preeclampsia diagnosis to examine their knowledge about whether they were at greater risk of developing a list of health complications. The participants (*n* = 127) displayed high awareness about being at greater risk of developing hypertension later in life (98%) and being more susceptible to stroke and CVD (67%). However, 60% of the “aware” participants reported that they gained knowledge by doing their own research, while only 25% heard about their long-term risk from their obstetrician, about 13% from their general practitioner and 6% from their midwife. Despite most participants (about 95%) having had their blood pressure measured, a lower proportion reported serum cholesterol and/or glucose screening (about 41%), and fewer had received advice on various lifestyle risk factors (ranging from 2% for smoking and about 30% for weight management, exercise and healthy eating).

The one study addressing both women's and HCP's knowledge assessed how relationships between risk, pregnancy, and women's health are understood and acted upon. Five women were interviewed, and they reported being either minimally or not informed around diagnosis about long-term CVD risk after PE. Recurrence of PE in a subsequent pregnancy was addressed with two of the five interviewees. Only one was tested and counseled at 6 weeks postpartum. Women were unsure whether their pregnancy history was transmitted to their family physician. Women did not always trust the skills and knowledge base as well as the decisions made by their care givers. Finally, participants had made extensive efforts to source information relating to their condition from places like the internet, online discussion boards, magazines, and even television series (12).

One of the qualitative studies in the UK showed that women with a family history of CVD disease had greater awareness of future CVD risk (29). Of the 12 women interviewed, seven had family history of CVD (29). Women without traditional risk factors found it hard to envisage themselves as being at risk and did not see the relevance of such information. The authors noted timing of discussions as an important element to consider when communicating about postpartum risk, taking into account situational factors of new motherhood, and when women are ready to consider their own health as well as their baby's, to engage successfully with this group of women (29). A study conducted in Portugal of 78 women with either history of PE or CH and superimposed PE showed that addressing of risk after PE by HCP predominantly did not happen (54 no vs. 24 yes) (16).

### Healthcare Provider's Knowledge

The studies about health providers' knowledge varied in the screening questions and detail of knowledge inquiry. Six studies addressed HCP's knowledge (including the study which addressed both groups) (12, 24–28). The studies assessed knowledge with varying depth, therefore the results display different aspects of knowledge.

A Canadian study with 554 participants (obstetrician/gynecologist, midwives, and family physicians) (26) showed that almost two-thirds (64%) knew that women with a history of gestational hypertension had a higher risk of developing hypertension in the future. About one-half of the clinicians were aware that women with a history of PE were at higher risk than nulliparous women to develop hypertension in the future. The study did not compare knowledge between the different HCP groups.

In one of the USA studies (28), participants were asked about their typical counseling for CVD risk reduction. This showed that of 161 participants (118 internists/internal medicine physicians, 53 obstetricians) 95% of internists and 70% of obstetricians reported providing general CVD risk reduction counseling. When asked about knowledge of future cardiovascular risks in women with preeclampsia, the majority in both groups were incorrect or unsure about the risk of several future comorbidities associated with a history of PE. With the exception of risk of future hypertension, where only 6% internists and 17% of obstetricians answered incorrectly, the comorbidities that were surveyed yielded a significant knowledge gap. The other risks surveyed were future risk of ischemic heart disease (56% internists and 23% obstetricians answered incorrect/unsure), stroke (48% internists and 38% obstetricians answered incorrect/unsure), and a shorter life expectancy which displayed the highest percentage of incorrect/unsure answers (79% internists and 77% obstetricians). Only 5% of internists and 42% of obstetricians asked about PE as part of taking a woman's medical history. Of the doctors asking about a PE history, only a small group (9% of internists and 38% of obstetricians) provided counseling to women at risk. The findings suggested that clinicians are not aware of the association between adverse pregnancy outcomes such HDP and CVD (28).

Wilkins-Haug et al. (27) undertook an anonymous survey in the USA. The survey was case-based, had 124 participants and explored obstetrician/gynecologist vs. internists' recognition of long-term CVD risk after preeclampsia. One aspect of the survey was to assess the participants' understanding of how pregnancy history may influence long-term cardiovascular risk, where information was collected through a combination of direct query, multiple choice responses, case-based questions, and branch logic. The second aspect assessed their general knowledge of CVD risk. Overall, about 28 and 15% of internists and obstetricians, respectively, indicated they would not obtain a pregnancy history when specifically assessing a patient's history for cardiovascular risk. When history of PE was obtained, internists were more likely to order fasting glucose test than gynecologists (48% vs. 21%).

In Germany, a survey about knowledge of the association between preeclampsia and long-term risks of CVD was distributed to a random sample of 500 obstetrician-gynecologists with 121 participating. Overall, the doctors with better knowledge of existing guidelines had better understanding of risks and were more likely to offer counseling to women with a history of PE. More specifically, 87% of doctors knew of the association between PE and future hypertension, whilst 79% knew about the association with stroke risk. Although the majority of the respondents were aware of the increased CVD risk post preeclampsia, the awareness of existing guidelines on long term follow up care and counseling of affected women remained deficient. Only 45% of participants were aware of these guidelines, however knowledge was higher amongst these participants (25).

The only study in a low to middle income country (LMIC) was undertaken by Adekanle et al. (24) in south western Nigeria. A survey was distributed to 146 healthcare professionals as part of a workshop at a teaching hospital. The majority (87%) were knowledgeable about future hypertension risk after PE and about ischemic heart disease (63%), stroke (69%), and kidney disease risk (73%). Forty-six percent counseled on CVD risk after hypertensive disease. The doctors had better knowledge (78% overall) than both nurses (58%) and community healthcare workers (54%). However, the majority (64%) were not aware that a shorter life expectancy is linked with preeclampsia, while only 38 (26%) asked about preeclampsia on routine visits and 46% counseled on cardiovascular risk.

#### Enablers and Barriers to Knowledge Acquisition for Healthcare Providers

A Canadian study (26) identified weaknesses in knowledge base and communication amongst the maternity care providers and community health care (family physicians). There was a significant discrepancy when addressing the communication between the hospital to community handover after HDP. Of the participating maternity care providers, 83% stated that they informed the family physician with regards to the woman's history after HDP. However, only 58% of the participating family physicians stated that they received HDP information about the women transitioning back into the community. Furthermore, only 12% of family physicians stated that they were made aware that women post HDP are at increased risk of CVD, despite 41% of maternity HCP claiming that this happened. This study suggests that effective identification and follow-up of women with HDP is not occurring.

A follow up Canadian study assessed whether HCP shared information with women about their increased CVD risk (12). In this study of 8 healthcare practitioners, three of the eight did not inform women of increased risk more than 50% of the time. Interviews were undertaken to explore participants' perceptions of and attitudes toward the relationship between PE and CVD risk. Structural, practical, and ideological barriers were shown to impede knowledge sharing between health care providers and women about the relationship between preeclampsia and CVD risk (12). Patient electronic records were not consistently available to all HCP, hence the community health care providers are reliant on written records transferred to them. One obstetrician in Hird et al.'s study (12) relied on assumed knowledge of midwives and family physicians to link PE to long-term CVD risk. HCP reported filtering what they said and when about a certain situation. Some were cautious about the timing (e.g., in high stress situation) and others felt that if the women did not ask they would probably not want to know and hence the HCP would not address the topic.

Three of the six studies mentioned guidelines (12, 24, 25). In Adekanle et al. (24) it is unclear which guideline participants are asked about. Their discussion refers to a national guideline, however declares that PE guidelines are institution based. Only 16% of participants were aware of a guideline, the authors suggest this number may reflect the medical practitioners as there was a smaller number of them. Heidrich et al. (25) asked about awareness of current national guidelines which comment on follow up of PE and future CVD risk management. Overall only 45% knew about these guidelines with significantly more knowledge in the group with guideline awareness. The group with guideline knowledge counseled women more frequently about long term risks, more frequently assessed blood pressure, had better knowledge of the link between HDP and CVD and screened for family history of PE more frequently. The third study (12) found that the absence of clinical practice guidelines had a possible effect on the postpartum management of PE and CVD risks. The current guidelines' focus was more on the diagnosis and the intrapartum management of HDP.

## Discussion

This scoping review found that, in most studies, women's and HCP's knowledge about the increased risk of CVD after HPD was low. The various studies explored differing aspects of knowledge. Some studies included one question about knowledge of the association of HDP and CVD, whereas other studies used further questions to differentiate amongst the various aspects of knowledge on this topic. Three studies used the term “risk perception” which showed a distinction between basic factual knowledge vs. how a woman at risk may perceive her own risk as true or not true. Issues with communication between different HCP (between hospital and community) as well as between HCP and women was identified, particularly when asking about pregnancy history when CVD risk assessing, transferring pregnancy history and risk factors to community health care providers, and counseling women on the long-term CVD risk. Due to the diversity in explored aspects of knowledge within the included studies it is difficult to compare and contrast the studies themselves. A common ground however is found in their discussion of enablers and barriers to the acquisition of knowledge.

### Women

#### Enablers to Acquisition of and Action on Knowledge

There were a number of enabling features for knowledge acquisition. The internet and access to a variety of information via online communities and networks appeared to be an enabler for women. Where women displayed reasonably high aspects of knowledge, it was found that had sourced this information by conducting their own research (12, 23). Women felt it was beneficial to receive information on how this risk could be reduced (29).

Clarification on the extent to which a history of preeclampsia and gestational hypertension are an independent factor for future CVD are considered helpful in the provision of effective communication (29). Interestingly, risk perception of HDP recurrence and future CVD due to HDP was higher in women who had a family history of CVD and/or PE with severe features (15, 29), further indicating that many other HDP affected women do not identify with the increased risk. They may not be aware that they could be affected in the future and are less likely to seek information as a result. Poor self-reporting of a PE and GH history may also accentuate the issue (30–33).

Some women felt that having access to other women who have had a similar pregnancy experience with HDP may have been a supportive move toward risk reducing lifestyle changes. Access to community of women who have experienced HDP or similar support groups may be helpful in feeling less isolated, more informed and supported (13, 14, 29). The thought of being able to support other women in similar situations was a motivator. The suggestion to have privacy maintained in this online community and a moderator who could validate medical information exchanged as well as keep the community's communication positive. In other areas of women's healthcare, including breastfeeding and polycystic ovarian syndrome, online community groups have been effective (34, 35) for women's emotional support and empowerment, so could be explored further for post-HDP women.

Online tracking of weight and blood pressure was deemed to be helpful in the application of knowledge with regards to lifestyle changes. Having support from family members when implementing lifestyle changes was deemed important and their family also benefiting from the healthy changes was a further motivator (14). Women felt that a reminder by their health care providers to follow up after HDP was needed to bring the mother's health back into mind after having given birth and transitioning to parenthood (29).

#### Barriers to Acquisition of and Action on Knowledge

A lack of knowledge from their health care provider on the link between HDP and CVD was a barrier to women, as well as their poor insight into or lack of action toward risk reducing lifestyle changes. The lack of knowledge about the link is also a barrier for women to then act on possible modifiable CVD risks or to simply gain insight into and understanding of the HDP they experienced. Barriers to action for women were predominantly related to family and caregiving responsibilities, lack of knowledge, lack of appropriate, and timely follow up as well as remembering what type of follow up and monitoring they needed, as well as poor recovery postpartum (13, 29). The amount and type of information given at any particular healthcare encounter was considered to be a barrier (12).

From the HCP perspective, HCP felt some information needed to be repeated over multiple visits to be truly understood, and that limited consultation time required them to prioritize the type of information shared with women. Some only responded to questions raised by the women and withheld other information. In turn, women were not confident they are being given all the information they need postpartum to manage their risk (12). Transition from hospital-based obstetric care to primary community-based care was also a barrier for women (and a system level barrier) as was lack of health insurance (in the USA) (13).

### Healthcare Providers

Where comparisons between HCP groups were made, obstetricians had a higher level of knowledge than family physicians, internal medical specialists, midwives or community health workers (24, 26, 28). Knowledge and application into practice was demonstrated when guidelines were available, and their existence was known (25).

There was only one study conducted in a LMIC (24). In this setting (Nigeria), knowledge was higher amongst doctors compared with “lower cadre” health workers (examples cited in the included paper include associate nurses, community health workers). Although different contextually, this statement could be applicable to high income countries where community-based health care providers (such as general practitioners/family physicians/community nurses) are close to the community.

#### Enablers to Acquisition of and Action on Knowledge

Implementation and knowledge of guidelines may provide an enabling environment for better knowledge and application of this knowledge (25, 28). An example of positive influence of the implementation of guidelines can be found in the Netherlands (36), where an increase in counseling following preeclampsia occurred. This is likely to reflect an increase in education of gynecologists over time regarding cardiovascular risk, resulting in confidence addressing these concerns with the women in their care.

The type of specialty training in the HCP domain was a further enabler. When knowledge amongst professions was compared, obstetricians were more knowledgeable than family physicians and midwives for example (24, 27, 28). This may be linked to the training, scope of practice as well as exposure to women with HDP that this medical specialty has. More generally, although potentially linked to specialty training, Wilkins-Haug et al. (27) found that when there was better overall knowledge of CVD risk factors and screening knowledge, this was associated with greater knowledge of association of PE with later life CVD.

#### Barriers to Acquisition of and Action on Knowledge

Poor communication between hospital and community HCP was mentioned in almost all HCP papers (13, 24, 26, 28). In areas where knowledge was high this did not translate into action-taking on the reduction of long-term risk factors (24). There were also a number of examples of withholding or not sharing information from women during CVD counseling or post HDP counseling (26, 28). Even when providers know about the association of PE with later CVD they did not apply this knowledge when counseling women. Further information on what a history of HDP means in terms of increased future risk and follow-up needed was perceived as not frequently shared by maternity specialists (26). Here too the gap between the information sharing by maternity HCP and information reception by community HCP was significant (26), with participants' answers possibly reflecting the intention of communication rather than what actually occurs in daily practice.

Studies comparing latest clinical guidelines in different countries highlight the variations in clinical recommendations (most likely due to lack of high-level evidence base for what is effective post-HDP follow-up) and lack of cost effective follow up. Some authors suggested further studies take place in order to inform practice guidelines and optimize prevention strategies (37). These include adjustment of general CVD guidelines to include taking a pregnancy history (38–40) and associating HDP with the increased risk of developing CVD.

A large amount of research exploring various aspects of women's increased CVD risk with a history of HDP have suggested that education as well as addressing modifiable risk factors could be targeted for improving the short- and long-term sequela for women (8, 17). The literature shows that poor pregnancy history taking is common. Recent population research has found women are less likely than men to have their cardiovascular (CVD) risks (not including preeclampsia history) fully assessed, and less often have risks appropriately managed when they are assessed (41). The gap may occur due to a number of potential barriers. Firstly, at individual and social levels, physicians may not be aware or familiar with existing guidelines on best practice in this matter. Secondly, physicians and women may have the old misconception of CVD being a man's disease. Furthermore, the misconception of senior physicians may have been passed on to the younger generation of physicians. In addition to financial and time and resource constraints, it is likely that women have been disadvantaged in receiving appropriate CVD risk factor assessment. When assessments do happen, women also are less likely than men to have their risks managed appropriately. Therefore, an increased rate of assessment and management of CVD risks are needed are needed in women generally, over and above specific management after HDP (41).

Considering pregnancy history when assessing a woman's CVD risk may assist in targeted efforts to initiate risk-reduction strategies in women with a history of pregnancy complications and improve communication between women and health care providers as well as communication between maternity and community providers. This may help decrease the burden of CVD in women (40, 42) and minimize the need for women having to rely mainly on exploring resources online to educate themselves (23).

### Implications for Practice

Overall, there appears to be a knowledge gap in women and HCP on the association of HDP and risk of CVD. This gap could be narrowed and the information about this topic needs to be distributed in a suitable, accessible and targeted way.

The study that reflected reasonably high knowledge levels and counseling of women reasoned that the participants were aware of the existence of guidelines on the topic and showed application of these in practice (25). Guidelines are available (5) however their existence alone may prove to be insufficient. A potential education campaign of guideline awareness is one of the solutions, to encourage implementation of guidelines into practice.

Knowledge of HDP link to future CVD in both the HCP and women would be optimal in order to make progressive adjustments to potentially reduce the risk of future disease. Studies conducting research on lifestyle adjustments for women with a history of HDP have already been published in some countries. This shows attempts to reduce risk by applying the knowledge HCP and women have.

A specific follow up clinic may be an effective method of prevention of future risk for women (43, 44) as knowledge alone about risk may not translate into motivation or changes to lifestyle in order to reduce risk (45). There are early benefits of counseling about lifestyle modifications in order to prevent CVD in women with recent preeclampsia. Channeling women into appropriate health centers once pregnancy care is completed may be an enabling approach (43, 44). Careful pregnancy screening and appropriate escalation to the right health care provider, women's risk profile can be identified and addressed. Appropriate referral offers opportunity to determine effective treatments that can prevent the progression of hypertensive disorders in pregnancy and in turn reduce future CVD (43).

### The Gap Identified in the Literature

This review has identified some gaps in the literature. The lack of evidence and hence of clear guidance on how to provide information to women who have experienced HDP identifies one of these gaps. More specific aspects of knowledge on the topic need to be assessed in women and HCP such as more specific knowledge of individual risk factors. Furthermore, higher numbers of women and HCP in a variety of countries and healthcare settings could be assessed and contribute to a more in-depth insight into knowledge levels and also on possible targeted knowledge enhancing strategies. Despite having gained insight into some of the enablers to knowledge acquisition and application, little evidence has been collected addressing what form education should take.

### Strengths and Limitations

Strengths of the review include the comprehensive search strategy and scoping review by two independent reviewers according to PRISMA-ScR criteria. It provides an up-to-date evidence-base of the literature on the topic of women's and HCP's knowledge of cardiovascular health after hypertensive pregnancy. Our scoping review looked at both perspectives (women and HCP) and contextualized these findings amongst a larger context of CVD screening and prevention, enablers and barriers as well as from a primary health perspective.

The included literature is limited to English language. The methods of the research we included are diverse and different aspects of knowledge were examined amongst different HCP. Women's medical conditions examined also slightly varied from one paper to another. Having included international literature, cultural health context with access to healthcare are different, this makes the findings more difficult to compare and contextualize. Knowledge is contextual, and knowledge of risk factors and risk reducing behavior does not imply action on this knowledge. This aspect is hard to measure and by participation alone, this may already show a sign of bias to being receptive to knowledge and possibly motivated to make lifestyle changes. When planning knowledge transmission and action on health after HDP, it is important to consider the local context. This applies to the country's available health services, workforce, and scope of follow-up care postpartum. Despite the different settings of the included studies, there were several common themes around knowledge gaps, barriers, and enablers of acquisition of knowledge that were found in this review, including low knowledge among women and HCP of CVD after HDP, lack of communication of knowledge by HCP with higher knowledge (usually obstetricians) to HCP colleagues and women, and women's use of informal sources to gain knowledge. This suggests some generalizability regardless of context.

## Conclusion

In general, there is a lack of knowledge amongst HCP and women regarding CVD risks after HDP Where women had higher levels of knowledge, the information was often obtained informally rather than from HCP. Obstetricians were generally more aware than other professions of the HDP-CVD link, however did not necessarily communicate this knowledge to either women or other HCP. Awareness of risk factors may provide, in conjunction with further research on effective risk reduction methods, a unique opportunity to plan future screening and preventative health recommendations by primary health care providers, which currently appears to be insufficient in women with a history of HDP. Further country and context-specific research on current status of women's and HCP's knowledge is therefore necessary when creating educational strategies to address knowledge gaps after HDP.

## Author Contributions

HR, AH, and CH contributed to the conception and design of the review. HR led the review of the literature, the analysis, and wrote the first draft. GL was the second reviewer of the literature, contributed to the analysis and drafting, and designed the tables, figures, and appendixes. All authors contributed to drafts and revising of the paper and approved the final version.

### Conflict of Interest

The Co-editor of the article collection of “Hypertension During Pregnancy and Future Risk of Cardiovascular and Other Long-Term Health Outcomes,” AH, is also a co-author of the submitted paper. However, AH played no role in the decision to peer review or ultimately accept this paper due to being a co-author. This was managed by Dexter Canoy, Associate Editor to avoid any perception or reality of conflict of interest. The remaining authors declare that the research was conducted in the absence of any commercial or financial relationships that could be construed as a potential conflict of interest.
