# Supplementary material for: Assessing Knowledge Gaps of Women and Healthcare Providers Concerning Cardiovascular Risk After Hypertensive Disorders of Pregnancy—A Scoping Review
Source: Front Cardiovasc Med. 2019 Nov 29;6:178. doi: 10.3389/fcvm.2019.00178 (PMC6895842; doi:10.3389/fcvm.2019.00178)
Supplement: Supplementary file 1 [file Data_Sheet_1.docx]

ANNEXES

**Assessing knowledge gaps of women and healthcare providers concerning cardiovascular risk after hypertensive disorders of pregnancy - A Scoping Review**

**Supplementary Table 1: Inclusion and exclusion criteria**

| **Inclusion criteria** | **Exclusion criteria** |
| --- | --- |
| Studies assessing women’s knowledge on increased CVD^a^ risk after HDP^b^ (PE^c^, HELLP^d^ and Eclampsia, GH^e^, CH^f^ with superimposed PE) | Studies assessing CH in pregnancy only |
| Full text available with detailed results | Only abstract is available e.g. conference, |
| Qualitative and quantitative studies, all methods, sample type and size | Reviews, trial registrations |
| Papers available in English language | Papers only available in other language than English |
| Original research | Papers without novel research |
| All years of publication | Studies on application of lifestyle modifications to reduce CVD risk |

^a^ Cardiovascular disease

**^b^** Hypertensive disorder of pregnancy

^c^ Preeclampsia

^d^ Hemolysis, elevated liver enzymes, low platelet count in pregnancy

^e^ Gestational hypertension

^f^ Chronic hypertension

**Supplementary Figure 1: PRISMA Flowchart of screening and selection process**

Full-text articles excluded, with reasons n=18

(n = 4 no full-text available

n = 2 not original research

n = 2 described planned future research

n = 0 not available in English

n = 10 did not specifically ask women or healthcare providers about knowledge of CVD risk after HDP)

Additional records identified through other sources n=15
(Snowball search n = 15)

Records identified through database searching n = 1452

(Embase n = 58, Medline n=56, Scopus n=761, ProQuest n=574, Cochrane n=2, PsycInfo n=1)

Full-text articles assessed for eligibility
(n = 30)

Records after duplicates removed
(n = 1210)

## Identification

## Included

## Eligibility

## Screening

Studies included in the qualitative synthesis
(n = 12)

Records excluded at title and abstract stage
(n =1176)

Inclusion/Exclusion Criteria Applied

**Supplementary Table 2: Characteristics of the included literature**

| Author(s),  Year,  Country, Publication Title | Study Objective | Study design | Timing of study  (Assessing women’s knowledge only) | Sample | Main Findings | Enablers of knowledge acquisition and action on knowledge | Barriers of knowledge acquisition and action on knowledge |
| --- | --- | --- | --- | --- | --- | --- | --- |
| Women’s knowledge | | | | | | | |
| Brown et al.  (1)  2013  United Kingdom  Women's perception of future risk following pregnancies complicated by preeclampsia | Determine women’s understanding of cardiovascular risk following a pregnancy complicated by PE^a^ | QUALITATIVE  Semi-Structured Interviews | At 5.8 – 19 months post PE | Women with history of PE  n=12 | - Family history of CVD^b^ associated with increased understanding of future cardiovascular risk - 4 women with first degree relatives with CVD and 3 with HT^c^ were more likely to recall their increased risk - 5 women did not recall future risk being discussed in clinic - 10 women stated they were not the “typical stereotype of a person at risk of heart problems” | - Planning future pregnancy - Family history CVD - Healthy home for family | - Lack of knowledge - Caregiving responsibilities |
| Seely et al.  (2)  2013  United States of America  Risk of future cardiovascular disease in women with prior preeclampsia: a focus group study | To assess level of knowledge regarding link between PE and CVD, motivators and barriers to lifestyle change and interest in a lifestyle modification program in women with a history of PE | QUALITATIVE  Focus Group | $\leq$ 5 years after pregnancy with PE | Women with history of PE  n=20 | - “Majority” had no prior knowledge of link between PE and CVD - “Few” said clinician had discussed long-term CVD risk. “Few” knew about link from online reading - “Majority” said PE not fully explained during pregnancy. “Many” indicated post-partum period was a good time to incorporate lifestyle changes | - Knowledge of risk - Desire to stay healthy for family - Access to community of women also affected by PE | - Lack of time. - Cost of healthy foods - Family responsibilities - Transition from obstetric care to primary care |
| Viana Pinto et al. (3)  2014  Portugal  Preeclampsia and future cardiovascular risk: are women and general practitioners aware of this relationship? The experience from a Portuguese Centre | To evaluate the impact of PE on the modification of lifestyle habits and decreasing cardiovascular risk factors in women at least 6 months following PE | CQUANTITATIVE  Survey | Pregnancy  $>$ 6 months ago | Women with PE and with CH with super-imposed PE  n=78 | - 31% of the women stated that their basic healthcare provider had addressed the item preeclampsia and future cardiovascular risks. - Almost 58% assessed blood pressure at least monthly - 28% undertook aerobic exercise at least weekly - Significant reduction in weight in women with superimposed PE - 23% women had one appointment per year with their healthcare provider |  | Lack of follow-up care structure |
| Skurnik et al.  (4) 2016  United States of America  Improving the postpartum care of women with a recent history of preeclampsia: a focus group study | To investigate barriers and facilitators towards learning about link between CVD and PE and engaging in lifestyle modifications to reduce this risk | QUALITATIVE Focus Group | At $\leq$ 6 months of experiencing PE | Women with a history of PE  n=14 | - 71% women were unaware of link between PE and future cardiovascular disease - 100% had access to primary health care provider - “Most” said healthcare providers did not explain link between PE and CVD | - Tracking behavior change - Support groups with other women - Health of family as a motivator | - Lack of knowledge - Lack of social support in lifestyle changes - Feeling of isolation post PE |
| Traylor et al.  (5) 2016  United States of America  Risk perception of future cardiovascular disease in women diagnosed with a hypertensive disorder of pregnancy | To evaluate a woman’s risk perception for future CVD following HDP at hospital discharge and at 2 weeks postpartum | QUANTITATIVE  Survey | Survey 1: prior to hospital discharge  Survey 2: at 2 weeks post discharge | Women with a history of PE and CH^d^  n=146  (PE no severe features n=76, PE with severe features n=41, CH n=29) | - A moderate or high risk perception of hypertension in later life was more likely to be reported by those with severe PE (65%) and chronic hypertension (75%) than those with PE without severe features (43%) - Approximately 70% of women believed that having had HDP^e^, exercising regularly, or controlling weight gain had NO effect on their risk for future CVD - More than 75% of women correctly identified that smoking and high cholesterol increased the risk of cardiovascular disease - Women delivering before 37 weeks were more likely to correctly identify an increased risk for future HDP, chronic hypertension, myocardial infarction, and stroke in their lifetime. They were also more likely to express a decreased desire for a future pregnancy compared with women delivering at term | - More severe PE and/or preterm delivery | - Lack of knowledge - Lack of professional support after delivery - Higher rates of depressive symptoms |
| Hutchesson et al., (6)  2018  Australia  Are women with a recent diagnosis of preeclampsia aware of their cardiovascular disease risk? A cross-sectional survey | To determine in women with prior PE, their awareness of future CVD risk, and the extent of CVD risk factor screening and advice offered since their diagnosis | QUANTITATIVE  Survey | Within $\leq$2 years of experiencing PE | Women with a history of PE  n=127 | - 96% knew of higher risk of hypertension, 67% for stroke and 66% for other CVD - Of those aware of raised CVD risk, 60% found out from own research, 25% their obstetrician^f^, 13% their GP^g^ and 6% their midwife - 95% had their blood pressure measured, and 41% had cholesterol or glucose measured |  |  |
| Healthcare provider’s knowledge | | | | | | | |
| MacDonald et al., (7)  2007  Canada  Hypertensive Disorders of Pregnancy and Long-Term Risk of Hypertension: What Do Ontario Prenatal Care Providers Know and What Do They Communicate? | To determine the knowledge base of Ontario maternity care providers regarding future health risks of GH and PE, and the communication of these risks | QUANTITATIVE  Survey | N/A | Total healthcare providers  n=554  (obstetricians n = 241, midwives n=139,  family physicians n=174) | - 58% of family physicians informed by maternity care providers of patients with hypertensive pregnancy - 83% of maternity care providers say they inform family physicians |  |  |
| Young et al., (8) 2012  United States of America  Physicians' knowledge of future vascular disease in women with preeclampsia | To determine if primary care providers were aware of the association between PE and future CVD, and whether they were providing appropriate counselling. | QUANTITATIVE  Survey | N/A | Internists n=118 and obstetricians n=53 | - “Majority” of physicians were unaware of the association between PE and future CVD - Internists and Obstetricians were unaware of ischemic heart disease risk (56% & 23% respectively), stroke risk (48% and 38% respectively) and reduced life expectancy (79% and 77% respectively) - 9% internists & 38% obstetricians counselled on CVD risk in women with PE. - 5% internists and 42% obstetricians included PE in history | - Implementation of guidelines | - Lack of knowledge of HDP link to CVD in healthcare providers - Lack of communication between healthcare providers - Inconsistencies in clinical education |
| Heidrich et al.,  (9)  2013  Germany  Preeclampsia and long-term risk of cardiovascular disease: what do obstetrician-gynaecologists know? | To assess whether obstetrician-gynaecologists are aware of the association between PE and maternal long-term adverse outcomes and providing appropriate counselling | QUANTITATIVE Survey study of Obstetricians (n=212) | PE |  | - 86.6% physicians knew of link between PE and future hypertension - 79% knew about association with stroke risk - 76% aware of lower life expectancy - Physicians with knowledge of guidelines regarding PE (45%) had increased understanding of risk and are more likely to offer counselling - 94% included recommendations for lifestyle changes in counselling strategy. - Blood pressure measurement 3 months’ post-partum (per guidelines) was undertaken by 76% of gynaecologists in outpatient settings | - Knowledge of guidelines |  |
| Adekanle et al.,  (10)  2015  Nigeria  Health workers' knowledge on future vascular disease risk in women with preeclampsia in South Western Nigeria | To assess the knowledge of health workers about the association between PE with future CVD and offering any risk-reduction counselling to women with PE | QUANTITATIVE Validated Survey undertaken at family planning clinics.  Physicians n=88, nurses/midwives n=19, community health workers n=39 | N/A |  | - 69% had knowledge of future risk of CVD following PE. - Medical doctors had better knowledge than both midwives and community health workers (78%, 58%, 54% respectively). - 46% of surveyed healthcare providers offered CVD risk-reduction counselling - 64% did not know of reduced life expectancy |  | - Lack of knowledge of healthcare providers - Type of healthcare profession - Differences in clinical training |
| Wilkins-Haugh et al.,  (11)  2015  United States of America  Recognition by women's health care providers of long-term cardiovascular disease risk after preeclampsia | To assess HCPs^h^ knowledge regarding pregnancy outcome as a risk factor for CVD and evaluate the factors associated with their responses to questions about routine surveillance for CVD | QUANTITATIVE Survey |  | Total n=124  Gynaecologists  n=49,  Internists  n=75 | - Gynaecologists were more likely to assess history of PE when undertaking CVD assessment than internists (73% vs 55%) - If history of PE, internists were more likely to order fasting glucose test than gynaecologists (48% vs. 21%) |  |  |
| Women’s knowledge and Healthcare provider’s knowledge | | | | | | | |
| Hird et al., 2017  (12)  Canada  Risk for cardiovascular disease after preeclampsia: differences in Canadian women and healthcare provider perspectives on knowledge sharing | To analyze women and HCPs perceptions of and attitudes towards the relationship between PE and CVD risk and assess how that is acted upon in Canadian healthcare | QUALITATIVE  Semi-Structured Interviews | Conducted 2 years after MacDonald study (with focus on HCP)  The timing after PE/pregnancy of the women is unclear | Total HCP n= 8 (Obstetricians n=5, GPs n=2, Midwives n=1)  Sourced from same sample recruited by the included HCP study by MacDonald et al. (2007).  Total women with history of PE n=5  Sourced from ‘former patients’ – unclear sample source | - 41% HCP did not inform patients of increased risk more than 50% of the time - Lack of guidelines and referrals were cited as concerns by healthcare professionals |  | - Urgency of situation prioritized over information. - Unreliable referrals and discharge management - No guidelines for post PE follow up - Appointments too brief - Focus on next pregnancy rather than future - Lack of knowledge (women) |

^a^ Preeclampsia

^b^ Cardiovascular disease

^c^ Hypertension

^d^ Chronic hypertension

^e^ Hypertensive disorder of pregnancy

^f^ obstetrician/gynaecologist

^g^ General practitioner

^h^ Healthcare provider

**Appendix 1: Preferred Reporting Items for Systematic reviews and Meta-Analyses extension for Scoping Reviews (PRISMA-ScR) Checklist**

| **SECTION** | **ITEM** | **PRISMA-ScR CHECKLIST ITEM** | **REPORTED ON PAGE #** |
| --- | --- | --- | --- |
| **TITLE** | | | |
| Title | 1 | Identify the report as a scoping review. | 1 |
| **ABSTRACT** | | | |
| Structured summary | 2 | Provide a structured summary that includes (as applicable): background, objectives, eligibility criteria, sources of evidence, charting methods, results, and conclusions that relate to the review questions and objectives. | 1-2 |
| **INTRODUCTION** | | | |
| Rationale | 3 | Describe the rationale for the review in the context of what is already known. Explain why the review questions/objectives lend themselves to a scoping review approach. | 3 |
| Objectives | 4 | Provide an explicit statement of the questions and objectives being addressed with reference to their key elements (e.g., population or participants, concepts, and context) or other relevant key elements used to conceptualize the review questions and/or objectives. | 3-4 |
| **METHODS** | | | |
| Protocol and registration | 5 | Indicate whether a review protocol exists; state if and where it can be accessed (e.g., a Web address); and if available, provide registration information, including the registration number. | N/A |
| Eligibility criteria | 6 | Specify characteristics of the sources of evidence used as eligibility criteria (e.g., years considered, language, and publication status), and provide a rationale. | 3-4 |
| Information sources* | 7 | Describe all information sources in the search (e.g., databases with dates of coverage and contact with authors to identify additional sources), as well as the date the most recent search was executed. | 3-4 |
| Search | 8 | Present the full electronic search strategy for at least 1 database, including any limits used, such that it could be repeated. | 3-4 |
| Selection of sources of evidence† | 9 | State the process for selecting sources of evidence (i.e., screening and eligibility) included in the scoping review. | 4 |
| Data charting process‡ | 10 | Describe the methods of charting data from the included sources of evidence (e.g., calibrated forms or forms that have been tested by the team before their use, and whether data charting was done independently or in duplicate) and any processes for obtaining and confirming data from investigators. | N/A |
| Data items | 11 | List and define all variables for which data were sought and any assumptions and simplifications made. | N/A |
| Critical appraisal of individual sources of evidence§ | 12 | If done, provide a rationale for conducting a critical appraisal of included sources of evidence; describe the methods used and how this information was used in any data synthesis (if appropriate). | 3-4 |
| Synthesis of results | 13 | Describe the methods of handling and summarizing the data that were charted. | 3-4 |
| **RESULTS** | | | |
| Selection of sources of evidence | 14 | Give numbers of sources of evidence screened, assessed for eligibility, and included in the review, with reasons for exclusions at each stage, ideally using a flow diagram. | 4 |
| Characteristics of sources of evidence | 15 | For each source of evidence, present characteristics for which data were charted and provide the citations. | 4 |
| Critical appraisal within sources of evidence | 16 | If done, present data on critical appraisal of included sources of evidence (see item 12). | 4-9 |
| Results of individual sources of evidence | 17 | For each included source of evidence, present the relevant data that were charted that relate to the review questions and objectives. | 4-9 |
| Synthesis of results | 18 | Summarize and/or present the charting results as they relate to the review questions and objectives. | 4-9 |
| **DISCUSSION** | | | |
| Summary of evidence | 19 | Summarize the main results (including an overview of concepts, themes, and types of evidence available), link to the review questions and objectives, and consider the relevance to key groups. | 9-13 |
| Limitations | 20 | Discuss the limitations of the scoping review process. | 14 |
| Conclusions | 21 | Provide a general interpretation of the results with respect to the review questions and objectives, as well as potential implications and/or next steps. | 14 |
| **FUNDING** | | | |
| Funding | 22 | Describe sources of funding for the included sources of evidence, as well as sources of funding for the scoping review. Describe the role of the funders of the scoping review. | 14--15 |

**Appendix 2: CASP Quality Appraisal of Included Studies**

| **Qualitative Research** | 1. Was there a clear statement of the aims of the research? | | 1. Is a qualitative methodology appropriate? | 1. Was the research design appropriate to address the aims of the research? | 1. Was the recruitment strategy appropriate to the aims of the research? | 1. Was the data collected in a way that addressed the research issue? | 1. Has the relationship between researcher and participants been adequately considered? | 1. Have ethical issues been taken into consideration? | 1. Was the data analysis sufficiently rigorous? | 1. Is there a clear statement of findings? | 1. How valuable is the research? | **Comments** | **Number of Criteria Met** |
| --- | --- | --- | --- | --- | --- | --- | --- | --- | --- | --- | --- | --- | --- |
| Brown et al. | **✓** | **✓** | | **X** | **✓** | **✓** | **✓** | **✓** | **✓** | **✓** | **✓** |  | 9/10 |
| Seely et al. | **✓** | **✓** | | **✓** | **CT** | **✓** | **X** | **✓** | **✓** | **✓** | **✓** |  | 8/10 |
| Skurnik et al. | **✓** | **✓** | | **✓** | **✓** | **✓** | **✓** | **X** | **✓** | **✓** | **✓** |  | 9/10 |
| Hird et al. | **✓** | **✓** | | **✓** | **✓** | **✓** | **CT** | **X** | **✓** | **✓** | **✓** |  | 8/10 |

**✓= yes, X = no, CT = cannot tell**

**Appendix 3: Search Terms used in electronic bibliographic databases**

| **Education, knowledge, barriers and Motivators** | **Hypertensive Disorders of Pregnancy** | **Risk of Cardiovascular Disease** |
| --- | --- | --- |
| Education  Education, Medical, Graduate  Education, Public Health Professional  Patient Education as Topic  Education, Nursing, Continuing  Education, Nursing, Graduate  Education, Nursing, Baccalaureate  Education, Nonprofessional  Nursing Education Research  Education, Medical  Education, Medical, Undergraduate  Education, Nursing  Education, Nursing, Associate  Education, Professional  Education, Continuing  Health Education  Education, Medical, Continuing  Education*  Educational intervention  Information dissemination  Knowledge sharing  Health Knowledge, Attitudes, Practice  Knowledge  Knowledge Management  Knowledge Bases  Risk Assessment  Knowledge gap  Awareness  Belief  Risk perception  Perception  Risk knowledge  Attitude of Health Personnel  Perspectives  Attitude* | Preeclampsia  Pre-Eclampsia  Gestational hypertension  Hypertension, Pregnancy-Induced | Risk identification  Risk Factors  Future cardiovascular disease  Later cardiovascular disease  Long-term cardiovascular risk |

1. Brown MC, Bell R, Collins C, Waring G, Robson SC, Waugh J, et al. Women's perception of future risk following pregnancies complicated by preeclampsia. Hypertens. 2013;32(1):60-73.

2. Seely EW, Rich-Edwards J, Lui J, Nicklas JM, Saxena A, Tsigas E, et al. Risk of future cardiovascular disease in women with prior preeclampsia: A focus group study. BMC Pregnancy and Childbirth. 2013;13:240.

3. Viana Pinto P, Rei M, Machado AP, Montenegro N. Preeclampsia and future cardiovascular risk: are women and general practitioners aware of this relationship? The experience from a portuguese centre. Obstetrics and gynecology international. 2014;2014:531539.

4. Skurnik G, Roche AT, Stuart JJ, Rich-Edwards J, Tsigas E, Levkoff SE, et al. Improving the postpartum care of women with a recent history of preeclampsia: a focus group study. Hypertens. 2016;35(3):371-81.

5. Traylor J, Chandrasekaran S, Limaye M, Srinivas S, Durnwald CP. Risk perception of future cardiovascular disease in women diagnosed with a hypertensive disorder of pregnancy. Journal of Maternal-Fetal and Neonatal Medicine. 2016;29(13):2067-72.

6. Hutchesson M, Shrewsbury V, Park F, Callister R, Collins C. Are women with a recent diagnosis of pre-eclampsia aware of their cardiovascular disease risk? A cross-sectional survey. Australian and New Zealand Journal of Obstetrics and Gynaecology. 2018;58(6):E27-E8.

7. MacDonald SE, Walker M, Ramshaw H, Godwin M, Chen X-k, Smith G. Hypertensive Disorders of Pregnancy and Long-Term Risk of Hypertension: What Do Ontario Prenatal Care Providers Know and What Do They Communicate? Journal of Obstetrics and Gynaecology Canada. 2007;29(9):705-10.

8. Young B, Hacker MR, Rana S. Physicians' Knowledge of Future Vascular Disease in Women with Preeclampsia. Hypertens. 2012;31(1):50-8.

9. Heidrich M, Wenzel D, von Kaisenberg C, Schippert C, von Versen-Höynck F. Preeclampsia and long-term risk of cardiovascular disease: what do obstetrician-gynecologists know? BMC Pregnancy and Childbirth. 2013;13:61.

10. Adekanle DA, Adeyemi AS, Olowookere SA, Akinleye CA. Health workers' knowledge on future vascular disease risk in women with pre-eclampsia in south western Nigeria. BMC Res Notes. 2015;8:576.

11. Wilkins-Haug L, Celi A, Thomas A, Frolkis J, Seely EW. Recognition by women's health care providers of long-term cardiovascular disease risk after preeclampsia. Obstetrics and Gynecology. 2015;125(6):1287-92.

12. Hird MJ, Yoshizawa RS, Robinson S, Smith G, Walker M. Risk for cardiovascular disease after pre-eclampsia: differences in Canadian women and healthcare provider perspectives on knowledge sharing. Health Sociology Review. 2017;26(2):128-42.
